# Identification of plasma biomarkers for discrimination between tuberculosis infection/disease and pulmonary non tuberculosis disease

**DOI:** 10.1371/journal.pone.0192664

**Published:** 2018-03-15

**Authors:** Marco Pio La Manna, Valentina Orlando, Paolo Li Donni, Guido Sireci, Paola Di Carlo, Antonio Cascio, Francesco Dieli, Nadia Caccamo

**Affiliations:** 1 Central Laboratory of Advanced Diagnosis and Biomedical Research (CLADIBIOR), Azienda Universitaria Ospedaliera Policlinico P. Giaccone, Palermo, Italy; 2 Dipartimento di Scienze Economiche, Aziendali e Statistiche, Università di Palermo, Palermo, Italy; 3 Dipartimento di Biopatologia e Biotecnologie Mediche, Università di Palermo, Palermo, Italy; 4 Department of Sciences for Health Promotion and Mother-Child Care “G. D’Alessandro”, University of Palermo, Palermo, Italy; King's College London, UNITED KINGDOM

## Abstract

We used the Luminex Bead Array Multiplex Immunoassay to measure cytokines, chemokines and growth factors responses to the same antigens used for RD1-based Interferon γ Release Assay (IGRA) test. Seventy-nine individuals, 27 active TB, 32 latent infection subsets, 20 individuals derivative purified protein (PPD) negative (subjects that do not have any indurative cutaneous reaction after 72 hrs of intradermal injection of PPD) and with other pulmonary disease were retrospectively studied. Forty-eight analytes were evaluated by Luminex Assay in plasma obtained from whole blood stimulated cells. The diagnostic accuracies of the markers detected were evaluated by ROC curve analysis and by the combination of multiple biomarkers to improve the potential to discriminate between infection/disease and non infection. Among 48 cytokines, 13 analytes, namely IL-3, IL-12-p40, LIF, IFNα2, IL-2ra, IL-13, b-NGF, SCF, TNF-β, TRAIL, IL-2, IFN-γ, IP-10, and MIG, were significantly higher in the active TB and LTBI groups, compared to NON-TB patients, while MIF was significantly lower in active TB patients compared to NON-TB and LTBI groups. The diagnostic accuracies of the markers detected in the culture supernatants evaluated by ROC curve analysis revealed that 11 analytes (IL2, IP10, IFN-γ, IL13, MIG, SCF, b-NGF, IL12-p40, TRAIL, IL2 Ra, LIF) discriminated between NON-TB and LTBI groups, with AUC for all analytes ≥0.73, while 14 analytes (IL2, IP10, IFN-γ, MIG, SCF, b-NGF, IL12-p40, TRAIL, IL2Ra, MIF, TNF-β, IL3, IFN-α2, LIF) discriminated between NON-TB and active TB groups, with AUC ≥0.78, that is a moderate, value in terms of accuracy of a diagnostic test. Finally, the combinations of seven biomarkers resulted in the accurate prediction of 88.89% of active TB patients, 82.35% of subjects with latent infection and 90% of non-TB patients, respectively. Taken together, our data suggest that combinations of whole blood Mycobacterium tuberculosis (Mtb) antigen dependent cytokines production could be useful as biomarkers to determine tuberculosis disease states when compared to non TB cohort.

## Introduction

Tuberculosis (TB) continues to be the cause of death of 4000 people per day [1], and the World Health Organization (WHO) and all the people that are involved in the field are still searching solutions able to control and prevent the socio-economic and medical problems caused by this pathology. In 2014, the WHO approved a new strategy to reach the ambitious target of ending the global TB epidemic by 2035 [2, 3]: the End TB Strategy. The goal will be reached when TB related deaths and active TB disease incidence, compared to the data of 2015, will be reduced by 95% and 90%, respectively, with a decrease in incidence less than 10 per 100, 000 population [4, 5]. Therefore, the discovery, development, and rapid implementation of new diagnostic tools have been identified as important components of the End TB strategy of WHO [6, 7].

One of the limits of the available diagnostic tests is that it is not possible to discriminate infection from disease. Therefore, the search for simple and cheap diagnostic methods with the aim to discriminate patients with active disease from subjects with latent infection, with the objective to cure patients with active disease and to block the spread of the pathogen from subjects with latent infection that are at high risk to develop the disease and that can cause the transmission of infection to healthy subjects. The identification of biomarkers that are detectable using peripheral blood with simple technologies represents a crucial aspect in the field, due to the limit of the IGRA tests commercially available as diagnostic tests, that are not able to discriminate between infection and active disease. In order to bypass these limitations, new techniques, including transcript microarrays, flow cytometry of intracellular cytokines, and multiplex microbead-based immunoassay (Luminex assay) of cytokines, have recently been introduced [8–12]. Moreover, these cytokines/chemokines were analyzed as a single biomarker or in combination, in order to find a tool to discriminate, in an unequivocal way, subjects with latent infection from that with active disease. In these studies, some biomarkers were found at the high significant difference between patients with active disease and LTBI subjects; while in other studies other cytokines have been indicated in order to discriminate the infection from disease, demonstrating that several factors, other than the pathogen, could influence the cytokines/chemokines levels [13–15].

In the current study, on the basis of the WHO End TB strategy, we have evaluated the levels of different cytokines and chemokines from plasma samples obtained from unstimulated and or antigen specific stimulated cells with the objective to discriminate active TB cases from latently infected contacts, or if the combination of different biomarkers could be useful as biosignature of disease/infection.

## Materials and methods

### Study participants

Seventy-nine individuals were retrospectively studied as here reported: ***(a)*** active TB group: 27 individuals diagnosed with TB (either with a positive culture for Mtb from *sputum* (48%) or with positive genexpert MTB/RIF, Mtb DNA resistance to rifampicin (52%) amplification results from biopsy specimens and/or biological fluids) 21 men, 6 women (age range 17–82 years) who started specific treatment within 8 days before enrolment; ***(b)*** latent infection subsets (LTBI): 32 individuals (24 men, 8 women, age range 17–84 years) who reported household or equivalent close contact (work) with smear-positive pulmonary TB patients in the previous 3 months, tested PPD positive (when the diameter was major of 10 mm) second ATS guidelines and QuantiFERON Gold In-Tube (QFT IT), Qiagen, -positive(IFN-γ levels ≥ 0.35UI/ml), with negative chest radiographic results for pulmonary lesions and no prior preventive therapy; ***(c)***other non-TB pulmonary infections (NON-TB): 20 individuals PPD negative (12 men, 8 women, age range 24–76 years); ***(d)***healthy donors (HD): 20 individuals PPD negative (14 men, 6 women, age range 21–68 years).

Patients were recruited from the Department of Infectious Disease, University Hospital of Palermo. All patients were treated in accordance with Italian guidelines and received therapy for 6 months. Treatment was successful in all participants, all of whom completed the full course of anti-TB chemotherapy. None of the TB patients had evidence of HIV infection, or was being treated with steroid or other immunosuppressive or anti-tubercular drugs at the time of their first sampling. The study was approved by the Ethical Committee of the University Hospital in Palermo (approval number 13/2013), where the patients were recruited. Informed consent was signed by all participants. QFT, IT (Qiagen, Carnegie, VA, Australia).

QFT-IT was performed as indicated by the manufacturer. Briefly, whole blood was collected in the QFT-IT tubes (Nil Control, TB-Ag and Mitogen) and incubated at 37°C for 16–24 hours. Following incubation, samples were centrifuged and the plasma was used to measure the IFN-γ produced in response to Mtb antigens, phytohaemagglutinin (PHA) and the negative control. Data are presented as IU/ml of IFN-γ; the cut-off value for a positive test was 0.35 IU/ml, according to manufacturer’s instructions.

### Luminex multiplex Immunoassay Bio-Rad

The concentrations of 48 host cytokines and chemokines, including biomarkers that have been already validated, were investigated in samples from all the study participants. Experiments were performed in a blinded manner, according to the instructions of the kit manufacturers.

The principle of the technique is based on developing color coded microspheres by combining different ratios of two dyes and this combination can give up to 100 different combinations, which enables measurement of 100 different analytes. The technology combines ELISA and flow cytometry together where the capture antibody is conjugated with beads or microspheres whereas the secondary antibody is conjugated with fluorochrome which quantifies the antigen- antibody reaction by measuring the relative fluorescence intensity. The assays were performed according to the supplier’s instructions. Briefly, following pre wetting of plates, 50 μl precombined beads of all the 48 individual cytokines or chemokines were added and washed twice.

Plasma samples (25 μl) were diluted 1:2 with the kit serum matrix and added to the plate. The plate was shaken for 30 s at 1000 RPM and then incubated for 1 h on plate shaken at 300 RPM at room temperature. The plates were washed twice and 25 μl of detection antibody was added per well and incubated for one hour on a plate shaker. Then strepatavidin-PE conjugate (50μl per well) was added and incubated for 30 min at room temperature. Finally, the plate was washed three times and 150 μl of sheath fluid was added to each well and then the plate was read on the Bio-Plex platform (Bio-Rad), with the Bio-Plex Software version 6.1 used for bead acquisition and analysis.

### QuantiFERON TB-gold in tube (QFT-IT)

QFT-IT (Cellestis Limited, Carnegie, Victoria, Australia) was performed using TB antigen-, mitogen- and unstimulated tube (nil). The tube containing TB antigen uses overlapping peptides from CFP-10 and ESAT-6 and TB7.7. QFT is a test for cell-mediated immune (CMI) responses to peptide antigens that simulate mycobacterial proteins. These proteins, ESAT-6, CFP-10, and TB7.7 (p4), are absent from all BCG strains and from most non tuberculous mycobacteria with the exception of M. kansasii, M. szulgai, and M. marinum. The assay was performed and the results were scored as indicated by the manufacturer's instructions (the cut-off value for a positive test was 0.35 IU/ml). An indeterminate score was assigned if the IFN-γ response to the mitogen after subtracting the nil IFN-γ response was <0.5 IU/ml or if the nil IFN-γ response was >8 IU/ml.

### Statistical analysis

Differences in the concentrations of host biomarkers detected in plasma samples from TB patients, LTBI subjects, NON-TB and HD were evaluated using the Kruskal- Wallis test distributed data. Biomarkers were compared between multiple groups using Kruskal Wallis tests. A non-parametric test able to compare more than two experimental groups. Dunn’s multiple comparison tests was used for post hoc correction to account for multiple comparisons.

Test performance in terms of sensitivity (ability of the test to identify the real positive subjects) and specificity (ability of the test to identify the real negative subjects) was evaluated for each cytokine by a ROC (Receiving Operating Characteristic) curve, the selective validation method of a quantitative diagnostic test in a population. The proportion of patients correctly diagnosed, that is the test accuracy, is proportional to the area under the curve (AUC), which can assume values between 0.5 (50% accuracy)and not accurate for AUC = 0.5, the test is poorly accurate for 0.5 <AUC≤0.7, the test is moderately accurate for 0.7 <AUC≤0.9, the test is highly accurate for 0.9 <AUC < 1 and the test is perfect for AUC = 1. The ROC curve also allows to identify the best cut off value that maximizes the difference between true positive subjects and false positives ones [16]. P-values <0.05were considered significant. The likelihood ratios was used for assessing the value of performing a diagnostic test.

General discriminant analysis (GDA) was used to evaluate the abilities of combinations of biomarkers to discriminate between Mtb-infected and uninfected groups or between active TB and LTBI. Optimal combinations of biomarkers were investigated by performing a best subsets analysis. All statistical analyses were performed and graphics were prepared using Statistica version 13 software (Statsoft, Ohio, USA), and GraphPad Prism software version 5.

## Results

### Measurement of host markers in LTBI subjects, TB patients and patients with other pulmonary infections

The baseline concentrations of host markers in TB patients were compared to the levels detected in subjects with LTBI and patients with pulmonary infections other than TB in order to avoid any unspecific background levels independently on mycobacterial stimulation. Initially, we scored those cytokine levels which were over the normal range concentrations, as calculated by the manufacturer in n = 66 healthy individuals. For comparison, we measured the 48 analytes in the serum of an independent cohort of n = 20 age- and sex-matched healthy individuals and found that reference values from the manufacturer were fully consistent with those from our independent cohort of healthy subjects (Wilcoxon p value = 0.26 for comparison of the two series of data). (Table 1).

**Table 1:** List of the cytokines, chemokines and growth factors evaluated by luminex assay Biorad (Germany) and their normal range assessed in healthy donors.

Analyte	pg/ml (Range)	pg/ml (Observed)	pg/ml (Median)	pg/ml (Mean)
IL-1α	0.50–1.40	0.40–1.40	0	0.12
IL-1β	<0.70	0.02–0.70	0	0.01
IL-1ra	6–665	0.20–665	23.94	42.01
IL-2	2–90	0.03–90	1.24	6.46
IL-2Ra	28–594	28–594	102.66	116.85
IL-3	13–170	13–170	41.06	44.54
IL-4	0.06–3	0.01–3	0	0.10
IL-5	1–7	0.01–7	0	0.15
IL-6	0.50–9	0.02–9	0	0.73
IL-7	0.60–13	0.01–14	0	0.27
IL-8	0.40–116	0.08–116	0	7.21
IL-9	2–500	0.38–500	19.40	37.50
IL-10	0.40–2	0.10–2	0	0.13
IL-12(p40)	36–646	36–646	0	60.19
IL-12(p70)	3–6	0.10–6	0	0.14
IL-13	0.80–9	0.01–9	0	0.33
IL-15	2–5	0.06–5	0	0.31
IL-16	10–1,270	10–1,270	77.50	94.79
IL-17	2–31	0.22–31	0	2.30
IL-18	9–812	9–812	68.05	75.71
IFN-α2	14–79	3.30–63	0	16.07
IFN-γ	7–124	0.60–124	8.68	13.43
TNF-α	6–98	0.10–98	0	5.92
TNF-β	1–13	0.71–13	0	0.31
TRAIL	8–272	8–272	66.61	65.91
CXCL1 (GRO-α)	9–365	9–365	22.35	36.33
CXCL9 (MIG)	86–7,911	86–7,911	289	617.05
CXCL10 (IP-10)	6–637	5.90–637	32.24	93.61
CXCL12 (SDF-1α)	8–92	8–92	0	13.79
CCL2 (MCP-1)	2–48	2–48	17.95	18.24
CCL3 (MIP-1α)	<2	0.01–2	0	0.15
CCL4 (MIP-1β)	5–47	1.70–47	11.24	14.75
CCL5 (RANTES)	100–2,282	100–2,282	0	203.64
CCL7 (MCP-3)	1–78	1–78	2.25	3.28
CCL11 (Eotaxin)	2–39	1.20–39	0	3.80
CCL27 (CTACK)	1–1,086	1–1,086	196	246.51
G-CSF	<1.50	<1.50	0	0.02
M-CSF	6–208	6–208	29.64	48.68
GM-CSF	3–122	0.80–122	6.78	12.47
SCF	16–837	16–837	167.57	172.88
SCGF-β	6,05–130,932	6,05–130,932	47,870	48,312
LIF	4–55	4–55	14.85	17.20
MIF	6–2,003	6–2,003	72.40	170.29
FGF-β	4–55	1.30–55	7.54	9
b-NGF	<1.10	<1.10	0	0.04
PDGF-BB	6–3,667	6–3,667	180.10	394.87
VEGF	0.50–9	0.01–9	0	0.43
HGF	63–1,868	63–1,868	195.20	255.24

The concentrations of 30 out of the 48 tested analytes were over the normal range in LTBI subjects and patients with active TB disease (Table 2). Baseline serum levels of 28 cytokines were found elevated in both groups, with percentages ranging from 40 to 100% in LTBI subjects and from 44 to 100% in TB patients. Baseline levels of no single analyte were differentially increased in sera from LTBI subjects. Conversely, baseline level of IFNα2 was slightly increased over the maximum normal range value (1,14-fold) in patients with active TB, but this was only detected in approximately one third of the patients, while the baseline of GM-CSF was increased in LTBI compared to active TB (1,20-fold) in the 50% of LTBI subjects.

**Table 2:** Medians and statistical analysis of the cytokines baseline levels.

							Dunn's multiple comparisons test		
	Non TB	LTBI	Active TB	Normal range	Kruskal-Wallis test		NIL NEG vs. NIL LTBI	NIL NEG vs. NIL ACTIVE TB	NIL LTBI vs. NIL ACTIVE TB
Analyte	% of patients upper normal range	% of patients upper normal range	% of patients upper normal range		p value		p value	p value	p value
Basic FGF	90%	78%	96%	1.30–55.00	0,0528	ns	>0,9999	0,1022	0,1197
Eotaxin	85%	88%	93%	1.20–39.00	0,5465	ns	>0,9999	>0,9999	0,851
G-CSF	100%	97%	100%	<1.50	0,1157	ns	0,7201	0,1141	0,8366
GM-CSF	45%	50%	48%	0.80–122.00	0,8664	ns	>0,9999	>0,9999	>0,9999
IFN-γ	40%	75%	70%	0.60–124.00	0,0537	ns	0,0519	0,2127	>0,9999
**IL-1β**	100%	97%	100%	0.02–0.70	0,0051	**	**0,0179**	**0,0077**	>0,9999
IL-1ra	20%	16%	15%	0.20–665.00	0,0562	ns	0,1565	0,0629	>0,9999
**IL-2**	10%	6%	4%	0.03–90.00	0,0341	*	0,1194	**0,036**	>0,9999
IL-4	85%	84%	93%	0.01–3.00	0,218	ns	0,253	0,6588	>0,9999
IL-5	5%	6%	0%	0.01–7.00	0,7723	ns	>0,9999	>0,9999	>0,9999
IL-6	100%	97%	100%	0.02–9.00	0,0934	ns	0,0917	0,4415	>0,9999
IL-7	30%	38%	26%	0.01–14.00	0,4545	ns	0,629	>0,9999	>0,9999
**IL-8**	100%	97%	93%	0.08–116.00	0,0161	*	0,5306	**0,0132**	0,2461
IL-9	20%	6%	22%	0.38–500.00	0,1278	ns	0,6297	0,1279	>0,9999
IL-10	100%	97%	100%	0.10–2.00	0,7698	ns	>0,9999	>0,9999	>0,9999
**IL-12(p70)**	100%	91%	96%	0.10–6.00	0,0062	**	**0,0167**	**0,01**	>0,9999
IL-13	20%	28%	35%	0.01–9.00	0,0867	ns	0,2386	0,0994	>0,9999
IL-15	55%	69%	56%	0.06–5.00	0,2735	ns	0,3651	>0,9999	0,8823
IL-17	95%	100%	100%	0.22–31.00	0,0779	ns	>0,9999	0,0819	0,4117
IP-10	80%	81%	89%	5.90–637.00	0,2025	ns	0,6522	>0,9999	0,277
**MCP-1 (MCAF)**	95%	97%	100%	2.00–48.00	0,0102	*	0,1554	**0,0075**	0,5804
MIP-1α	95%	97%	96%	0.01–2.00	0,1592	ns	0,2462	0,2905	>0,9999
MIP-1β	85%	69%	85%	1.70–47.00	0,4969	ns	0,7958	>0,9999	>0,9999
**PDGF–BB**	0%	0%	7%	6.00–3,667.00	0,0143	*	0,5805	**0,0123**	0,201
RANTES	85%	84%	81%	100,00–2.282,00	0,3143	ns	0,7307	0,4309	>0,9999
TNF-α	55%	81%	89%	0.10–98.00	0,051	ns	0,0652	0,1298	>0,9999
**VEGF**	100%	100%	100%	0.01–9.00	0,0298	*	**0,039**	0,0814	>0,9999
CTACK	60%	76%	100%	1.00–1,086.00	0,3238	ns	0,5438	0,5198	>0,9999
GRO-a	80%	94%	100%	9.00–365.00	0,6316	ns	>0,9999	>0,9999	> 0,9999
HGF	30%	24%	11%	63.00–1,868.00	0,5387	ns	>0,9999	0,8739	>0,9999
IFN-α2	60%	59%	44%	3.30–63.00	0,6583	ns	>0,9999	>0,9999	>0,9999
IL-1a	59%	100%	100%	0.40–1.40	0,4344	ns	>0,9999	0,8889	0,7695
IL-2Ra	20%	12%	11%	28.00–594.00	0,7087	ns	>0,9999	>0,9999	>0,9999
IL-3	100%	94%	100%	13.00–170.00	0,8275	ns	>0,9999	>0,9999	>0,9999
IL-12(p40)	80%	71%	89%	36.00–646.00	0,5405	ns	>0,9999	>0,9999	0,8275
IL-16	0%	0%	0%	10.00–1,270.00	0,131	ns	0,1413	0,377	>0,9999
IL-18	10%	6%	6%	9.00–812.00	0,2328	ns	0,2645	0,7535	>0,9999
**LIF**	80%	82%	94%	4.00–55.00	0,048	*	>0,9999	**0,0751**	0,1916
MCP-3	70%	82%	94%	1.00–78.00	0,2451	ns	>0,9999	0,3016	0,9322
M-CSF	40%	53%	28%	6.00–208.00	0,6022	ns	>0,9999	>0,9999	0,9684
MIF	80%	65%	94%	6.00–2,003.00	0,263	ns	>0,9999	0,3635	0,7695
MIG	60%	18%	50%	86.00–7,911.00	0,1655	ns	0,174	0,7619	>0,9999
b-NGF	100%	82%	94%	<1.10	0,4464	ns	0,7495	>0,9999	0,9538
SCF	0%	0%	0%	16.00–837.00	0,7475	ns	>0,9999	>0,9999	>0,9999
SCGF-b	0%	0%	0%	6,054.00–130,932.00	0,1127	ns	0,2636	>0,9999	0,2024
SDF-1a	100%	94%	100%	8.00–92.00	0,9409	ns	>0,9999	>0,9999	>0,9999
TNF-β	30%	47%	33%	0.71–13.00	0,8992	ns	>0,9999	>0,9999	>0,9999
TRAIL	0%	0%	6%	8.00–272.00	0,6917	ns	>0,9999	>0,9999	>0,9999

Median baseline levels of cytokines analyzed in the three groups, Kruskal-Wallis test with Dunn’s correction.

* p<0.05

**p<0.01.

The bold cytokines show higher concentrations than the normal range.

*Fr*. is the frequency of subjects with a higher cytokine concentration than the normal range.

To understand whether increased levels of several different analytes were a feature of TB infection/disease or were found also in other pathologic conditions, we measured their baseline concentrations in sera from patients with other non-TB pulmonary infections (n.20). As reported in Table 2, 29 out of 30 analytes were increased both in sera of TB patients and in NON-TB patients over the normal range, suggesting that elevated baseline levels of these molecules reflect an active infection/inflammation state, irrespective of the etiology.

**Fig 1:**
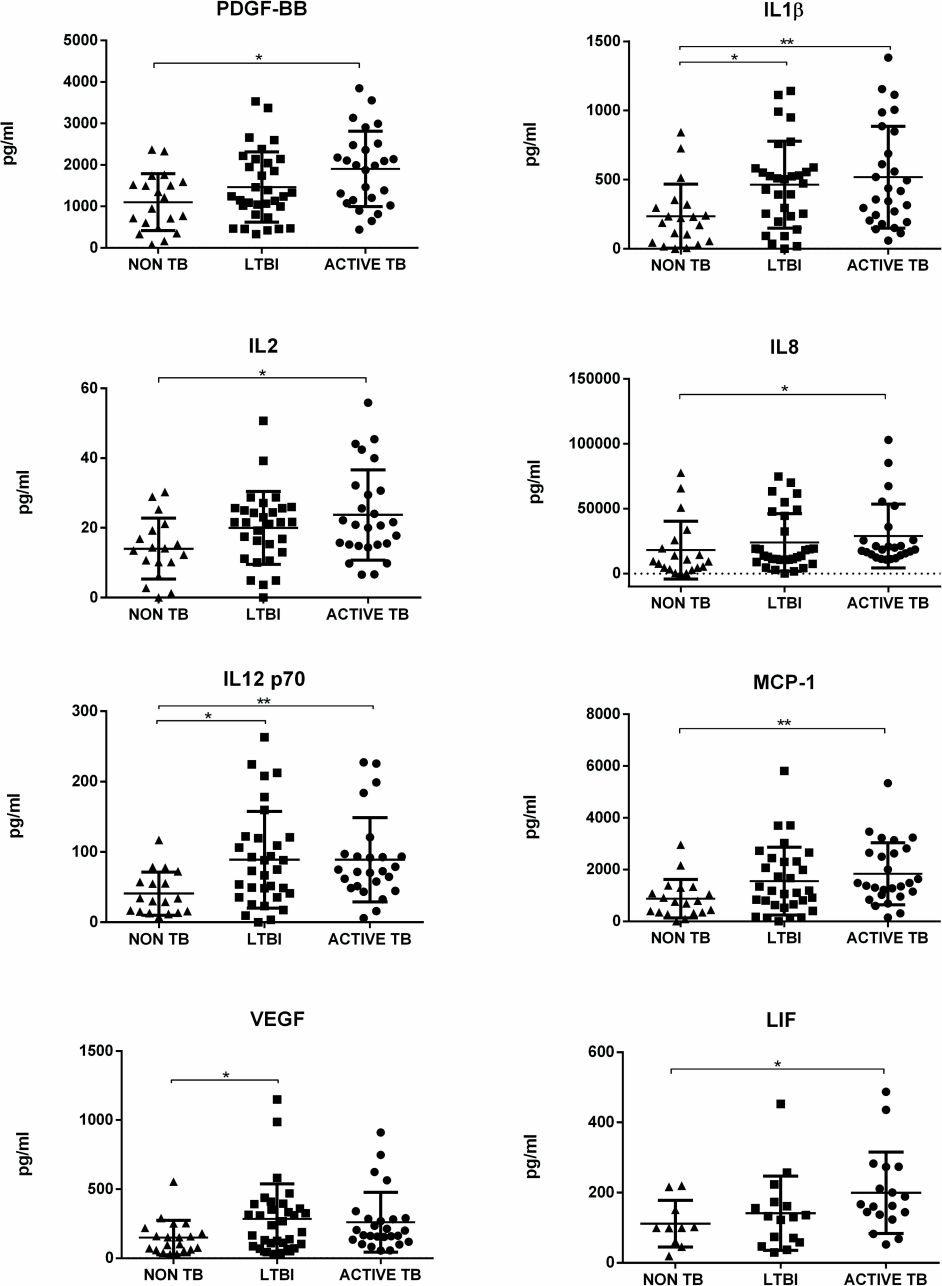
Scatter plots showing biomarker concentrations in unstimulated supernatants from participants with non TB disease (triangles), LTBI (squares), active TB patients (circle). Biomarkers were measured by Luminex assay. Median concentrations are indicated with horizontal bars. Statistical differences were analyzed using Kruskal-Wallis test with Dunn’s correction. *p<0.05, ** p<0.01.

To check for potential biomarkers capable of differentiating between LTBI subjects and patients with active TB, and between these latter and patients with other non-TB pulmonary infections, we compared the median baseline analyte concentrations in the tested LTBI, TB and NON-TB groups by Kruskal-Wallis tests. Results show (Table 2) that median baseline levels of 7 analytes, namely IL-1β, IL2, IL-8, IL-12p70, MCP-1, PDGF-BB, VEGF and LIF were significantly different among LTBI subjects, TB and NON-TB patients; furthermore, after post-test correction using Dunn’s multiple comparison tests, the median concentrations of IL-1β, IL12p70 and VEGF were significantly higher in LTBI subjects compared to NON-TB patients, and the median concentrations of PDGF-BB, IL-1β, IL-2, IL-8, IL12p70, MCP-1 and LIF were significantly higher in TB patients compared to NON-TB patients (Fig 1).

Of note, however, median baseline concentrations of the 48 tested analytes did not significantly differ between LTBI subjects and patients with active TB, indicating that there was no baseline biomarkers capable to discriminate between LTBI and active TB disease.

**Fig 2:**
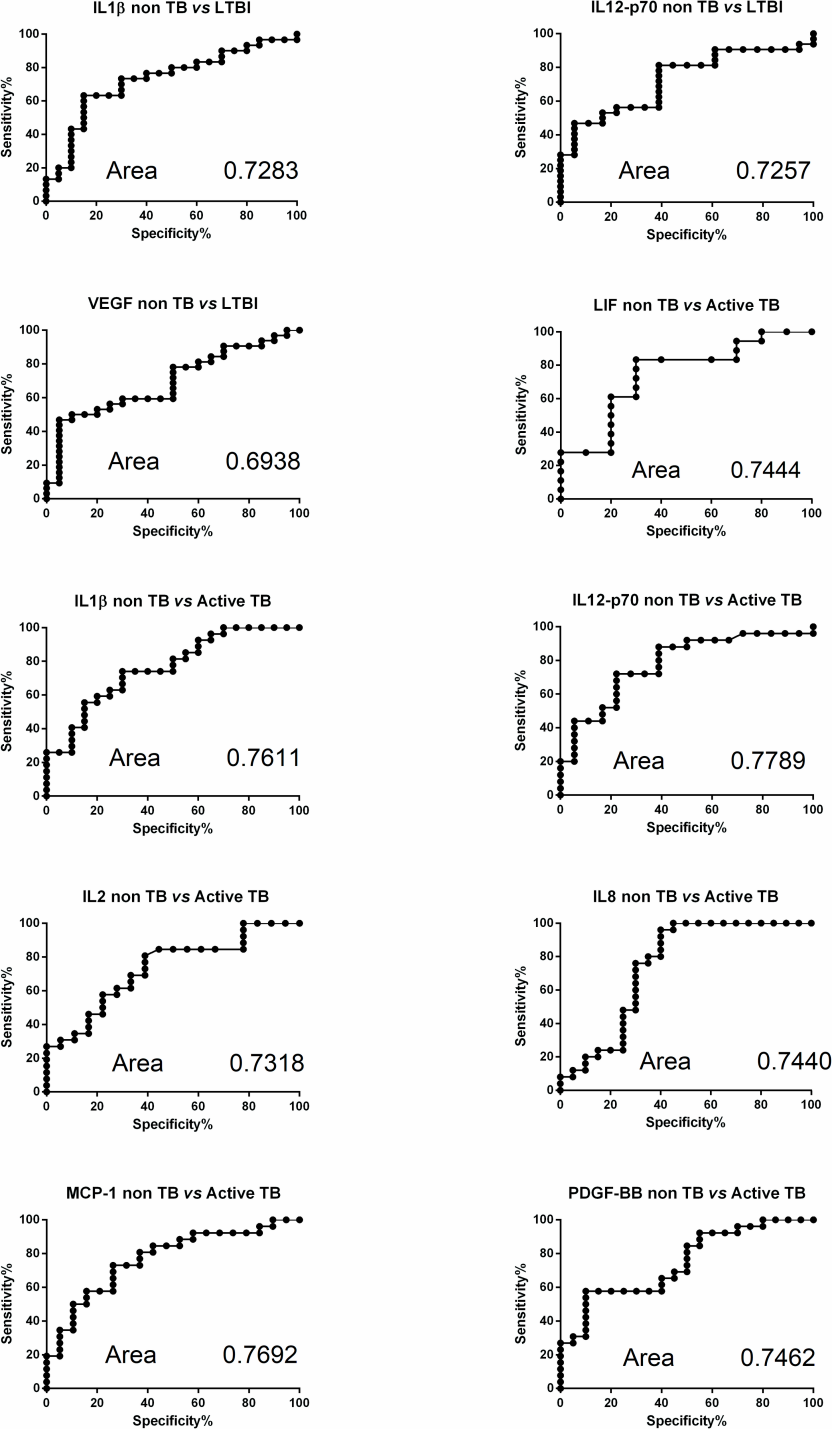
Receiver operating characteristic (ROC) curves for the baseline cytokine values comparing LTBI subjects and patients with active TB with non TB patients. The solid line shows the result of absolute values of each biomarker. The area under the curve (AUC) is indicated.

When the diagnostic accuracies of individual host markers were investigated by ROC curve analysis, the area under the ROC curve (AUC) was ≥ 0.70 for the 8 markers found significantly increased in LTBI subjects and active TB patients (Table 3). These markers have proven to be very effective in distinguishing non TB from active TB. At their optimal unstimulated cut off values, IL-8, IL-12p70 and MCP-1 had a sensitivity of 72%, 76% and 73% respectively, and IL-1β, PDGF-BB and LIF had a specificity of 85%, 90% and 80% respectively. The best performance characteristic was with unstimulated IL12p70 with a sensitivity of 73% and specificity of 78% (Table 3). Representative plots showing the 10 individual baseline host markers with the best diagnostic accuracy are shown in Fig 2.

**Table 3:** ROC curves features of markers increased in LTBI and Active TB.

	**ROC curve of non TB *vs* LTBI**									
	Area under the ROC curve	Std. Error	95% confidence interval	*Pvalue*	cutoff	Sensitivity%	95% CI	Specificity%	95% CI	Likelihood ratio
IL-1β	0.7283	0.0734	0.5844 to 0.8722	0.00669	> 373.1	63.33	43.86% to 80.07%	85	62.11% to 96.79%	4.222
IL12 p70	0.7257	0.07212	0.5843 to 0.8671	0.008629	> 72.69	53.13	34.74% to 70.91%	83.33	58.58% to 96.42%	3.188
IL-2	0.6713	0.08274	0.5091 to 0.8335	0.04891	> 17.20	63.33	43.86% to 80.07%	72.22	46.52% to 90.31%	2.28
IL-8	0.6161	0.08502	0.4495 to 0.7828	0.1649	> 10992	67.74	48.63% to 83.32%	60	36.05% to 80.88%	1.694
MCP-1	0.6613	0.07746	0.5094 to 0.8132	0.05765	> 1092	54.84	36.03% to 72.68%	73.68	48.80% to 90.85%	2.084
PDGF-BB	0.6094	0.08119	0.4502 to 0.7685	0.188	> 1230	53.13	34.74% to 70.91%	55	31.53% to 76.94%	1.181
VEGF	0.6938	0.07373	0.5492 to 0.8383	0.01972	> 260.9	50	31.89% to 68.11%	90	68.30% to 98.77%	5
LIF	0.5875	0.1156	0.3609 to 0.8141	0.4606	> 112.3	62.5	35.43% to 84.80%	70	34.75% to 93.33%	2.083
**ROC curve of non TB. *vs* Active TB**										
	Area under the ROC curve	Std. Error	95% confidence interval	*Pvalue*	cutoff	Sensitivity%	95% CI	Specificity%	95% CI	Likelihood ratio
IL-1β	0.7611	0.07001	0.6239 to 0.8984	0.002427	> 355.7	55.56	35.33% to 74.52%	85	62.11% to 96.79%	3.704
IL12 p70	0.7789	0.0725	0.6368 to 0.9210	0.002015	> 57.60	72	50.61% to 87.93%	77.78	52.36% to 93.59%	3.24
IL-2	0.7318	0.07671	0.5814 to 0.8822	0.009627	> 19.65	57.69	36.92% to 76.65%	77.78	52.36% to 93.59%	2.596
IL-8	0.744	0.08302	0.5812 to 0.9068	0.005345	> 14618	76	54.87% to 90.64%	70	45.72% to 88.11%	2.533
MCP-1	0.7692	0.07132	0.6294 to 0.9091	0.002252	> 1118	73.08	52.21% to 88.43%	73.68	48.80% to 90.85%	2.777
PDGF-BB	0.7462	0.07233	0.6044 to 0.8879	0.004583	> 1821	57.69	36.92% to 76.65%	90	68.30% to 98.77%	5.769
VEGF	0.7058	0.07795	0.5530 to 0.8586	0.01778	> 161.3	61.54	40.57% to 79.77%	70	45.72% to 88.11%	2.051
LIF	0.7444	0.1007	0.5469 to 0.9419	0.03494	> 155.7	61.11	35.75% to 82.70%	80	44.39% to 97.48%	3.056

### Measurement of mycobacterial antigen-specific host markers in LTBI subjects, TB patients and patients with other pulmonary infections

We compared the median analyte concentrations in the supernatants of mycobacterial Ag stimulated cells from LTBI, TB and NON-TB groups by Kruskal-Wallis tests. In order to exclude the contribution of unstimulated marker level to the diagnosis of TB infection/disease, for each study participant before the analysis of the data, the antigen specific responses of the different cytokines and chemokines were evaluated by subtraction of the unstimulated levels from the antigen-stimulated response.

**Fig 3:**
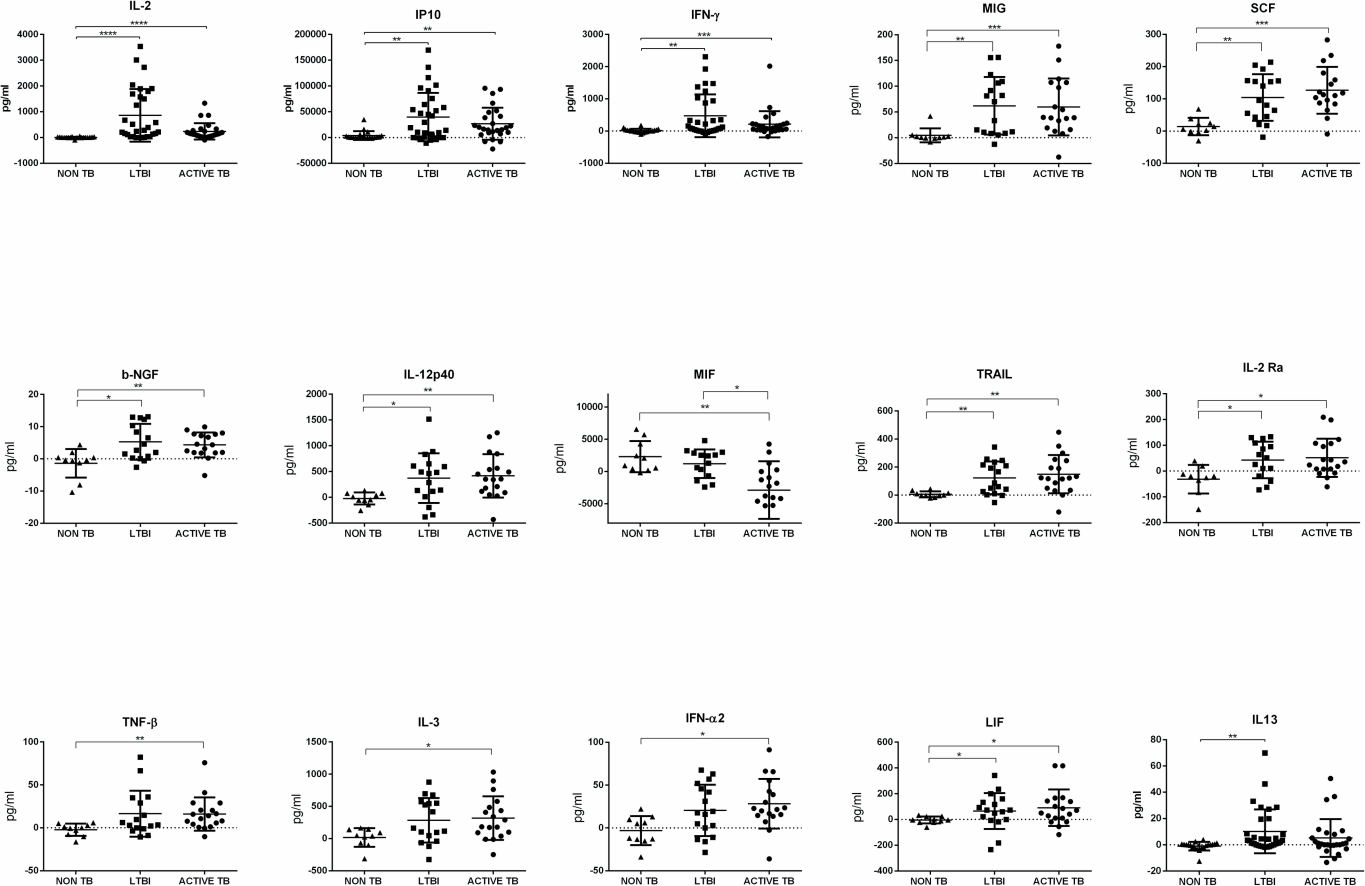
Scatter plots showing biomarker concentrations in antigen stimulated supernatants with subtracted Nil value from participants with non TB disease(triangles), LTBI (squares), active TB patients (circle). Biomarkers were measured by Luminex assay. Median concentrations are indicated with horizontal bars. Statistical differences were analyzed using Kruskall Wallis tests. *p<0.05, ** p<0.01.

Results show (Table 4) that in response to Mtb antigen specific stimulation by ESAT-6/CFP-10, median concentrations of 14 analytes, namely IL-2, IP-10, IFN-γ, MIG, SCF, b-NGF, IL-12-p40, MIF, TRAIL, IL-2ra, TNF-β, IL-3, IFNα2, LIF and IL-13 were significantly different in the active TB and LTBI groups, compared to NON-TB patients. Furthermore, after post-test correction using Dunn’s multiple comparison tests, the median concentrations of IL12-p40, IL-2ra, SCF, TRAIL, IL-2, IFN-γ, IP-10, b-NGF, LIF and MIG were significantly higher in the active TB and LTBI subjects compared to NON-TB patients, and the median concentrations of IFNα2, IL-3, and TNF-β were significantly higher in TB patients compared to NON-TB patients (Fig 3). Finally, the median concentrations of IL-13 was significantly higher only in LTBI compared to NON-TB patients. As with baseline levels of tested analytes, there were no statistically significant differences in median concentrations of the tested analytes in Mtb antigen-stimulated samples from LTBI subjects and patients with active TB, except for MIF that shows an appreciable and statistically significant decrease in the active TB group compared to LTBI and non TB groups.

**Table 4:** Medians and statistical analysis of cytokine levels after antigen specific stimulation with subtraction of baseline.

							Dunn's multiple comparisons test		
	Non TB	LTBI	Active TB	Normal range	Kruskal-Wallis test		Ag-NIL NEG vs. Ag—NIL LTBI	Ag-NIL NEG vs. Ag—NIL Act. TB	Ag—NIL LTBI vs Ag.-.NIL Act.TB
Analyte	% of patients upper normal range	% of patients upper normal range	% of patients upper normal range	pg/ml	p value		p value	p value	p value
Basic FGF	0%	0%	0%	1.30–55.00	0,3507	ns	0,4541	0,9515	> 0,9999
Eotaxin	0%	12%	4%	1.20–39.00	0,4187	ns	0,7966	0,6547	> 0,9999
G-CSF	50%	38%	36%	<1.50	0,2068	ns	0,2764	0,4318	> 0,9999
GM-CSF	0%	4%	0%	0.80–122.00	0,8174	ns	> 0,9999	> 0,9999	> 0,9999
**IFN-γ**	**10%**	42%	48%	0.60–124.00	0,0003	***	**0,0004**	**0,0027**	> 0,9999
IL-1β	45%	42%	64%	0.02–0.70	0,3585	ns	0,5116	> 0,9999	0,9416
IL-1ra	0%	16%	4%	0.20–665.00	0,2291	ns	> 0,9999	0,2896	0,72
**IL-2**	**0%**	69%	68%	0.03–90.00	< 0,0001	****	**< 0,0001**	**< 0,0001**	0,5463
IL-4	5%	15%	8%	0.01–3.00	0,311	ns	0,6375	> 0,9999	0,5554
**IL-5**	**0%**	8%	4%	0.01–7.00	0,0339	*	0,8795	0,3191	**0,028**
IL-6	70%	46%	44%	0.02–9.00	0,6	ns	> 0,9999	> 0,9999	> 0,9999
IL-7	15%	15%	12%	0.01–14.00	0,6539	ns	> 0,9999	> 0,9999	> 0,9999
IL-8	45%	42%	36%	0.08–116.00	0,3979	ns	> 0,9999	0,5293	> 0,9999
IL-9	0%	0%	0%	0.38–500.00	0,6374	ns	> 0,9999	> 0,9999	> 0,9999
**IL-10**	**30%**	15%	20%	0.10–2.00	0,0397	*	**0,035**	0,2389	> 0,9999
IL-12(p70)	15%	15%	16%	0.10–6.00	0,0421	ns	0,0672	0,0792	> 0,9999
**IL-13**	**0%**	23%	28%	0.01–9.00	0,0033	**	**0,0022**	0,1727	0,3893
IL-15	25%	42%	36%	0.06–5.00	0,6884	ns	> 0,9999	> 0,9999	> 0,9999
IL-17	25%	42%	36%	0.22–31.00	0,7224	ns	> 0,9999	> 0,9999	> 0,9999
**IP-10**	**45%**	69%	84%	5.90–637.00	0,0026	**	**0,0048**	**0,0088**	> 0,9999
MCP-1 (MCAF)	45%	81%	68%	2.00–48.00	0,4415	ns	0,6271	> 0,9999	> 0,9999
MIP-1a	40%	38%	32%	0.01–2.00	0,3835	ns	> 0,9999	0,5069	> 0,9999
MIP-1b	50%	31%	60%	1.70–47.00	0,0835	ns	0,9293	0,8343	0,0778
**PDGF–BB**	**0%**	0%	0%	6.00–3,667.00	0,0423	*	0,1245	**0,0486**	> 0,9999
RANTES	20%	23%	24%	100.00–2,282.00	0,7542	ns	> 0,9999	> 0,9999	> 0,9999
TNF-α	20%	35%	28%	0.10–98.00	0,79	ns	> 0,9999	> 0,9999	> 0,9999
**VEGF**	**15%**	8%	4%	0.01–9.00	0,0015	**	**0,0034**	**0,0045**	> 0,9999
CTACK	0%	0%	0%	1.00–1,086.00	0,5625	ns	0,9307	> 0,9999	> 0,9999
GRO-a	40%	59%	44%	9.00–365.00	0,7153	ns	> 0,9999	> 0,10000	> 0,10001
HGF	0%	0%	0%	63.00–1,868.00	0,1554	ns	0,1701	0,981	0,8104
**IFN-α2**	**0%**	12%	17%	3.30–63.00	0,0133	*	0,1087	**0,0104**	> 0,9999
IL-1a	30%	47%	33%	0.40–1.40	0,6236	ns	> 0,9999	0,9955	> 0,9999
**IL-2Ra**	**0%**	0%	0%	28.00–594.00	0,0195	*	**0,0401**	**0,0275**	> 0,9999
**IL-3**	**0%**	47%	67%	13.00–170.00	0,0419	*	0,1203	**0,0451**	> 0,9999
**IL-12(p40)**	**0%**	24%	22%	36.00–646.00	0,0037	**	**0,0133**	**0,0046**	> 0,9999
IL-16	0%	0%	0%	10.00–1,270.00	0,3716	ns	0,6493	> 0,9999	0,758
IL-18	0%	0%	0%	9.00–812.00	0,3452	ns	0,5321	0,6017	> 0,9999
**LIF**	**0%**	59%	50%	4.00–55.00	0,0346	*	0,0629	**0,0519**	> 0,9999
MCP-3	20%	53%	50%	1.00–78.00	0,1776	ns	0,1908	0,6126	> 0,9999
M-CSF	0%	6%	0%	6.00–208.00	0,3932	ns	0,8376	0,5487	> 0,9999
**MIF**	**40%**	47%	17%	6.00–2,003.00	0,0029	**	> 0,9999	**0,007**	**0,0208**
**MIG**	**0%**	53%	72%	86.00–7,911.00	0,0008	***	**0,0029**	**0,0013**	> 0,9999
**b-NGF**	**20%**	71%	89%	<1.10	0,0044	**	0,0102	0,0081	> 0,9999
**SCF**	**0%**	0%	0%	16.00–837.00	0,0004	***	**0,0051**	**0,0004**	> 0,9999
SCGF-b	10%	6%	0%	6,054.00–13,0932.00	0,3387	ns	> 0,9999	0,4237	> 0,9999
SDF-1a	20%	41%	39%	8.00–92.00	0,2878	ns	0,68	0,36	> 0,9999
**TNF-β**	**0%**	41%	50%	0.71–13.00	0,0098	**	0,0832	**0,0077**	> 0,9999
**TRAIL**	**0%**	6%	17%	8.00–272.00	0,0013	**	**0,0092**	**0,0013**	> 0,9999

Bold cytokines show appreciable and statistically significant differences between non-TB and LTBI or active TB

*p<0.05

**p<0.01

*** p<0.001.

**Fig 4:**
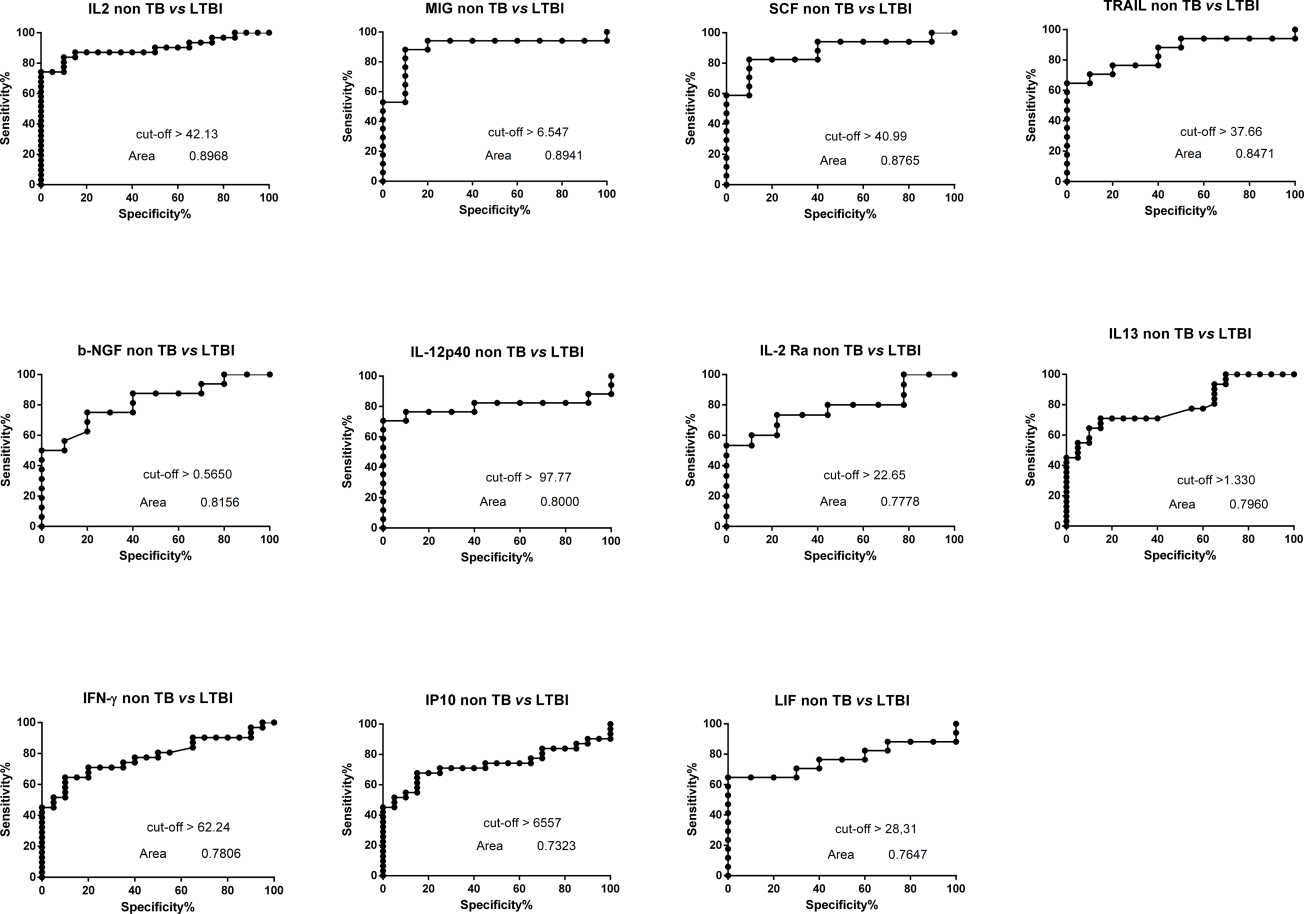
Receiver operating characteristic (ROC) curves for the cytokine values with subtracted Nil value comparing LTBI subjects with non TB patients. The solid line shows the result of the absolute values of each cytokine. The area under the curve (AUC) is indicated.

The biomarkers detected were analysed using receiver operating characteristic (ROC) curves, the test accuracy that is proportional to the area under the curve (AUC), which can assume values between 0.5 (50% accuracy) and 1 (100% accuracy). The ROC curve also allows to identify the best cut off value that maximizes the difference between true positive subjects and false positives ones. ESAT-6/CFP-10-specific levels of 11 analytes (IL2, MIG, SCF, TRAIL, b-NGF, IL12-p40, IL2Ra, IL13, IFN-γ, IP10 and LIF) discriminated between NON-TB and LTBI groups, with AUC ≥0.73. Out of these 8 markers, ESAT-6/CFP-10-specific level of MIG and SCF had the best sensitivity and specificity of >80% (Table 5 and Fig 4).

**Table 5:** ROC curves features of the 11 markers increased in LTBI.

	ROC curve of non TB *vs* LTBI (Ag-Nil)									
	Area under the ROC curve	Std. Error	95% confidence interval	*Pvalue*	cutoff	Sensitivity%	95% CI	Specificity%	95% CI	Likelihood ratio
IL-2	0.8968	0.04533	0.8079 to 0.9856	< 0.0001	> 42.13	74.19	55.39% to 88.14%	95	75.13% to 99.87%	14.84
IP10	0.7323	0.07094	0.5932 to 0.8713	0.005487	> 6226	67.74	48.63% to 83.32%	85	62.11% to 96.79%	4.516
IFN-γ	0.7323	0.07094	0.5932 to 0.8713	0.005487	> 64.24	64.52	45.37% to 80.77%	90	68.30% to 98.77%	6.452
IL-13	0.796	0.06165	0.6751 to 0.9168	0.000403	> 1.330	64.52	45.37% to 80.77%	90	68.30% to 98.77%	6.452
MIG	0.8941	0.06935	0.7582 to 1.030	0.000774	> 6.547	88.24	63.56% to 98.54%	90	55.50% to 99.75%	8.824
SCF	0.8765	0.06837	0.7424 to 1.011	0.00132	> 40.99	82.35	56.57% to 96.20%	90	55.50% to 99.75%	8.235
b-NGF	0.8156	0.08294	0.6530 to 0.9782	0.007802	> 0.5650	75	47.62% to 92.73%	80	44.39% to 97.48%	3.75
IL-12-p40	0.8	0.09117	0.6213 to 0.9787	0.01048	> 97.77	76.47	50.10% to 93.19%	90	55.50% to 99.75%	7.647
TRAIL	0.8471	0.07543	0.6992 to 0.9949	0.003067	> 37.66	70.59	44.04% to 89.69%	90	55.50% to 99.75%	7.059
IL-2 Ra	0.7778	0.09485	0.5918 to 0.9637	0.02539	> 22.65	60	32.29% to 83.66%	88.89	51.75% to 99.72%	5.4
LIF	0.7647	0.09289	0.5826 to 0.9468	0.0239	> 28.31	64.71	38.33% to 85.79%	90	55.50% to 99.75%	6.471

**Fig 5:**
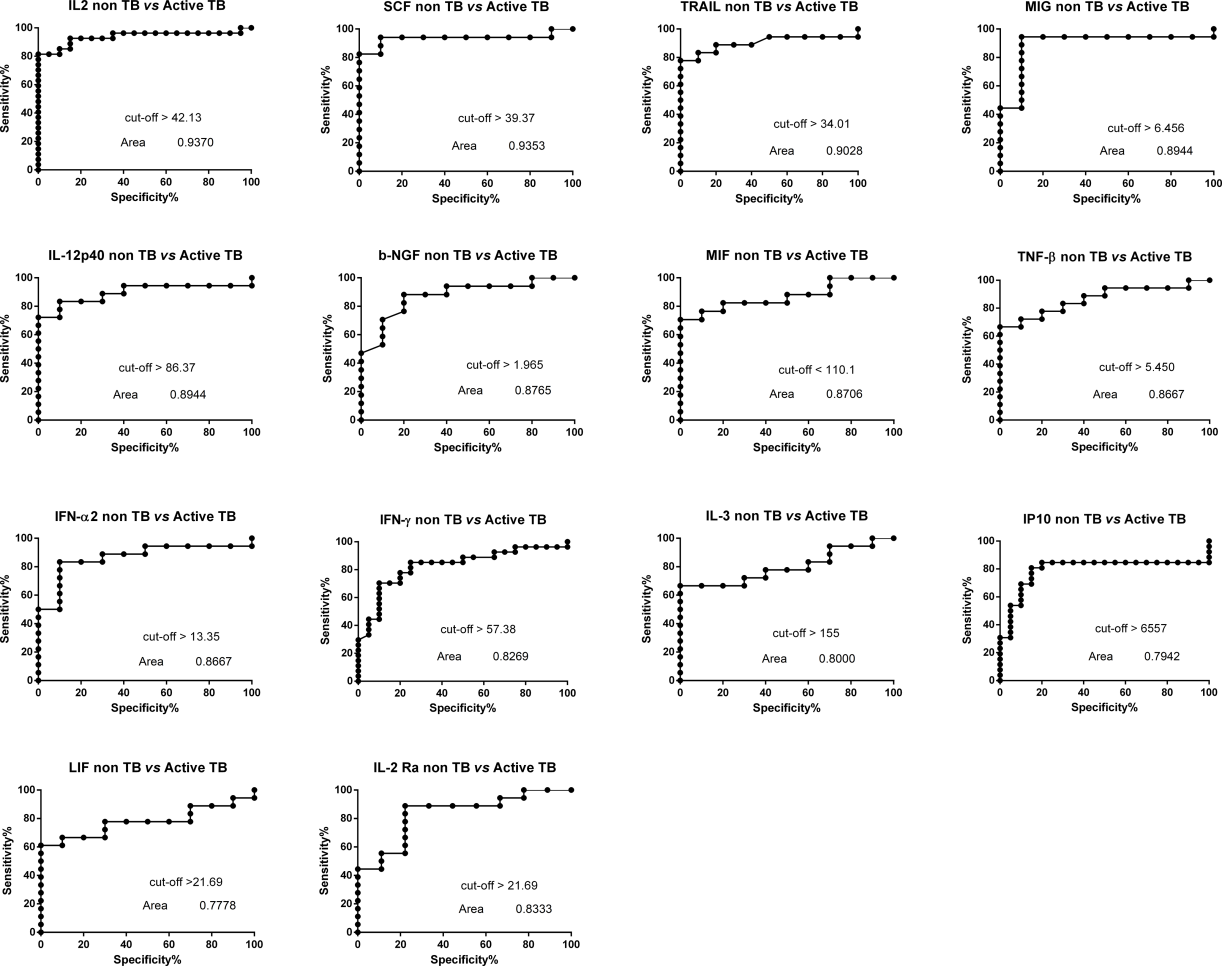
Receiver operating characteristic (ROC) curves for the cytokines values with subtracted Nil value comparing active TB patients with non TB patients. The solid line shows the result of the absolute values of each cytokine. The area under the curve (AUC) is indicated.

Similarly, when the diagnostic accuracies of the markers detected in the culture supernatants were evaluated by ROC curve analysis, ESAT-6/CFP-10-specific levels of 14 analytes (IL2, SCF, TRAIL, MIG, IL12-p40, b-NGF, MIF, TNF-β, IFN-α2, IFN-γ, IL3, IP10, LIF, IL2 Ra,) discriminated between NON-TB and active TB groups, with AUC ≥0.78. Two out of these 14 markers, ESAT-6/CFP-10-specific level of MIG and SCF had again the best sensitivity and specificity of >90% (Table 6 and Fig 5).

**Table 6:** ROC curves features of the 14 markers increased in active TB.

	ROC curve of non TB *vs* Active TB (Ag-Nil)									
	Area under the ROC curve	Std. Error	95% confidence interval	*p value*	cutoff	Sensitivity%	95% CI	Specificity%	95% CI	Likelihood ratio
IL2	0.7778	0.09485	0.5918 to 0.9637	0.02539	> 42.13	81.48	61.92% to 93.70%	95	75.13% to 99.87%	16.3
IP10	0.7942	0.07397	0.6492 to 0.9393	0.000704	> 6557	80.77	60.65% to 93.45%	85	62.11% to 96.79%	5.385
IFN-γ	0.8269	0.06213	0.7051 to 0.9487	0.000148	> 57.38	70.37	49.82% to 86.25%	90	68.30% to 98.77%	7.037
MIG	0.8944	0.07156	0.7542 to 1.035	0.000669	> 6.456	94.44	72.71% to 99.86%	90	55.50% to 99.75%	9.444
SCF	0.9353	0.05365	0.8301 to 1.040	0.000205	> 39.37	94.12	71.31% to 99.85%	90	55.50% to 99.75%	9.412
b-NGF	0.8765	0.06869	0.7418 to 1.011	0.00132	> 1.965	70.59	44.04% to 89.69%	90	55.50% to 99.75%	7.059
IL12-p40	0.8944	0.06315	0.7706 to 1.018	0.000669	> 86.37	83.33	58.58% to 96.42%	90	55.50% to 99.75%	8.333
TRAIL	0.9028	0.06148	0.7823 to 1.023	0.000513	> 34.01	83.33	58.58% to 96.42%	90	55.50% to 99.75%	8.333
IL2 Ra	0.8333	0.08273	0.6711 to 0.9955	0.005499	> 21.69	55.56	30.76% to 78.47%	88.89	51.75% to 99.72%	5
MIF	0.8706	0.06827	0.7367 to 1.004	0.00157	< 110.1	76.47	50.10% to 93.19%	90	55.50% to 99.75%	7.647
TNF-β	0.8667	0.06776	0.7338 to 0.9995	0.001563	> 5.405	72.22	46.52% to 90.31%	90	55.50% to 99.75%	7.222
IL3	0.8	0.08311	0.6371 to 0.9629	0.009651	> 155.0	66.67	40.99% to 86.66%	90	55.50% to 99.75%	6.667
IFN-α	0.8667	0.0723	0.7249 to 1.008	0.001563	> 13.35	83.33	58.58% to 96.42%	90	55.50% to 99.75%	8.333
LIF	0.7778	0.08809	0.6051 to 0.9505	0.01655	> 25.70	66.67	40.99% to 86.66%	90	55.50% to 99.75%	6.667

### General discriminant analysis (GDA)

To determine whether combinations of multiple biomarkers could improve the ability to detect Mtb-infected individuals, we examined the performance of all combinations of 23 biomarker responses (PDGF NIL, IL1β NIL, IL2 NIL, IL8,NIL, IL12p70 NIL, MCP-1 NIL, VEGF NIL, LIF NIL, IL2 Ag-NIL, IP10 Ag-NIL, IFN-γ Ag-NIL, MIG Ag-NIL, SCF Ag-NIL, b-NGF Ag-NIL, IL12-p40, MIF Ag-NIL, TRAIL Ag-NIL, IL2RaAg-NIL, IL13 Ag-NIL, TNF-β Ag-NIL, IL3 Ag–NIL, IFN-α2 Ag-NIL, LIF Ag-NIL) significantly differed between Mtb-infected cases (active TB n. 27, LTBI n. 32) and non TB group n. 20 (see also Figs 2 and 3). Using a GDA, all of the top–six or seven biomarker combinations (Table 7) accurately predicted active TB patients, LTBI subjects and non TB patients. In particular, seven biomarker combinations could predicted from 77.78 to 88.89% of active-TB patients, from 64.71 to 82.35% of LTBI subjects and from 90% to 100% of non TB patients. IL-2 was the biomarker most frequently included in the top seven-biomarker models, followed, in order, by IFN-γ, MIF, IL2Ra, LIF[Nil], followed by PDGF-BB [Nil], TRAIL and MCP-1[Nil] (Table 7).

**Table 7:** General discriminant analysis of different biomarker combinations.

GDA					Wilks' lambda	Active TB vs LTBI		Active TB vs Non TB		LTBI vs Non TB	
Seven biomarkers	Active TB (%)	LTBI (%)	Non TB (%)	Total (%)		*F*	*p*	*F*	*p*	*F*	*p*
MCP-1 _[NIL]_, LIF _[NIL]_, IL2, IFN-γ, MIF, TRAIL, IL2Ra	88.89	82.35	90.00	86.67	0.242	5.12	0.0004	6.15	<0.0001	4.83	0.0007
PDGF _[NIL]_, LIF _[NIL]_, IL2, IFN-γ, MIF, IL12-p40, IL2Ra	83.33	76.47	100.00	84.44	0.247	4.82	0.0007	6.62	<0.0001	4.81	0.0007
PDGF _[NIL]_, LIF _[NIL]_, IL2, IFN-γ, MIF, TRAIL, IL2Ra	77.78	64.71	100.00	77.78	0.243	4.71	0.0008	6.43	<0.0001	4.78	0.0007
Sixbiomarkers											
MCP-1 _[NIL]_, LIF _[NIL]_, IL2, IFN-γ, MIF, TRAIL	88.89	70.59	90.00	82.22	0.280	6.06	0.0002	6.31	0.0001	4.20	0.0026
MCP-1 _[NIL]_, LIF _[NIL]_, IL2, IFN-γ, MIF, L12-p40	83.33	70.59	80.00	77.78	0.279	5.71	0.0003	6.42	0.0001	4.52	0.0016
PDGF _[NIL]_, LIF _[NIL]_, IL2, IFN-γ, MIF, IL12-p40	77.78	64.71	100.00	77.78	0.278	5.55	0.0004	6.91	<0.0001	4.43	0.0018

The top six biomarker combinations could predicted from 77.78% to 88,89% of active TB patients, from 64,71 to 70,59% of LTBI subjects and from 80% to 100% of non TB patients. IL2, IFN-γ, LIF, MIF were frequently occurring biomarkers, followed by MCP-1[Nil], IL12-p40 and TRAIL. These results, clearly demonstrate that Mtb infection display a different combination of cytokines/chemokines expression, highlighting that the immune response is completely different between infection and non TB status.

## Discussion

In the present study, we have investigated the potential role of 48 cytokines/chemokines in plasma obtained from stimulated whole blood of adults, as biomarkers of infection/disease by comparing patients with active TB disease with LTBI subjects and with non-TB patients. We have shown that multiple biomarkers detected in the antigen stimulated supernatants can contribute to a diagnostic signature with the ability to discriminate between mycobacterial infected patients and non TB patients.

Several groups of research have focused their studies on assessing the quality of several biomarkers, that could have poor specificity and sensitivity when used alone, but which show a good performance when used in combination.

Preliminary data on the studies of biomarkers available in internet web such as PubMed, Embase, and Web of Science have reported either diagnostic accuracy and statistical significance of TB biomarkers able to identify active TB even if lacking of validation; in particular, for the majority of biomarkers (n = 399), diagnostic performance is not reported (161 biomarkers), or is based on testing of a non-blinded, usually retrospective set of conveniently obtained samples (170 biomarkers), or on blinded testing in a single study (68 biomarkers) as recently reported by Yerlikaya et al.[17].

From these studies, only 12 biomarkers have been confirmed in studies where these biomarkers have been evaluated prospectively, one of these biomarkers, has been evaluated as host biomarker and urine test has been approved by the WHO [18], but they require validation in a larger cohort of samples. Our data showed that the comparison of the median baseline of 7 analytes, namely IL-1β, IL2, IL-8, IL-12p70, MCP-1, PDGF-BB, VEGF responses significantly differed between Mtb-infected and non TB groups.

Several recent studies that have highlighted that various biomarkers explored in whole blood and peripheral blood mononuclear cells, investigated in adults or children, have been detected as alternative biomarkers for IFN-γ to detect Mtb-infected individuals [19–29]. In our study, cytokines, chemokines and growth factors showing a higher median concentration in active TB and LTBI compared to non TB, were not able to detect any differences between LTBI and active TB patients.

In contrast with our data, Anbarasu et al. have found an increase of PDGF in TB patients, compared with HD in response to M. tuberculosis culture filtrate proteins (CFPs), with other cytokines such as G-CSF, IL-1Ra, IL-6, IL-7, IL-8, IL-9 in endemic setting population, even if the study was limited to the low number of subjects analysed [29]. In fact, the increase of this cytokine probably is due to the different antigen used, very recently a distinct patterns of cytokines and chemokines has been demonstrated that are released in response to different innate ligands that interact with specific pattern recognition receptors[30].

The diagnostic accuracies of the different cytokines was evaluated by ROC curve analysis in order to discriminate the different study populations. We found that several cytokines were able to discriminate, with a good performance, LTBI from non TB. From this analysis, 11 cytokines showed a good performance with the area under the curve >0.8.

Similarly, from the analysis of the diagnostic accuracy of the markers evaluated between active TB patients and non TB patients, 13 markers were able to discriminate with a AUC≥ 0.78, with two markers MIG and SCF, able to reach the best sensitivity and specificity of 90%.

The concept of combining multiple biomarkers to improve the potential to discriminate between LTBI and active TB has also been explored previously.

Our data revealed that the six or seven-marker combinations correctly classified from 80 to 100% of non TB subjects, while could correctly identified from 77.78% to 88,89% of active TB patient and from 64–74% to 82,35% of LTBI subjects, highlighting that these combinations considerably improved the ability to discriminate between active TB and LTBI from non TB group, although the ability to predict non TB patients was found better reaching 90 of 100% of subjects who not have Mtb infection/disease.

Won et al. have investigated on the ability of eight Mtb antigen-specific biomarkers in the Mtb-infected group to be significantly different from those of the HCs, they have found that the combination of five Mtb-specific biomarkers and two unstimulated biomarkers, and one Mtb-specific biomarker ratio showed significant differences between active TB and LTBI. Three unstimulated biomarkers and 5 Mtb-specific biomarkers were significantly different between active TB and non TB groups [31]. Previous studies have reported that the combination of three cytokines, TNF-α, with IL-1Ra or IL-10 correctly classified 95.5% or 100% of cases, respectively [21]. Furthermore, Frahm et al. also reported that the combination of MCP-1 and IL-15 correctly classified 86.4% of cases [28]. Collectively, these findings suggest that combining cytokine biomarkers results in more accurate discrimination between Mtb and non TB infection.

In a recent study, serum levels of eotaxin, MIP-1α, sIL-2Rα, and lipocalin 2 were found different expressed in pulmonary tuberculosis patients *vs*. non TB patients [32]. From these markers, eotaxin was found to be the most accurate immunological biomarker, as evaluated by ROC curve analysis, that differentiated pulmonary tuberculosis and non TB patients. The power to differentiate between the different cohorts was increased by evaluating 4 different biomarkers in combination (eotaxin, MIP-1α, sIL-2Rα, and lipocalin 2). This study did not analyze these biomarkers as a biosignature of infection in LTBI subjects, limiting their use as diagnostic tools in discriminating between infection and disease.

In conclusion, our data add additional knowledge to the field of biomarkers that alone or in combination might further understanding of pulmonary tuberculosis infection/disease. A paucity of knowledge regarding specific biomarkers for pulmonary tuberculosis represents a big barrier to the development of diagnostic tests, specific treatment, and appropriate monitoring of disease. An additional large-scale study which target various Mtb-infected populations needs to be performed to validate the potential role of the different cytokines analyzed in detail in this study in order to validate their potential use as diagnostic biomarkers of Mtb infection.
